# Chemotherapeutic Sensitization of Leptomycin B Resistant Lung Cancer Cells by Pretreatment with Doxorubicin

**DOI:** 10.1371/journal.pone.0032895

**Published:** 2012-03-07

**Authors:** Chuanwen Lu, Changxia Shao, Everardo Cobos, Kamaleshwar P. Singh, Weimin Gao

**Affiliations:** 1 Department of Environmental Toxicology, The Institute of Environmental and Human Health, Texas Tech University, Lubbock, Texas, United States of America; 2 Department of Internal Medicine, Texas Tech University Health Sciences Center, Lubbock, Texas, United States of America; Faculty of Pharmacy, Ain Shams University, Egypt

## Abstract

The development of novel targeted therapies has become an important research focus for lung cancer treatment. Our previous study has shown leptomycin B (LMB) significantly inhibited proliferation of lung cancer cells; however, p53 wild type lung cancer cells were resistant to LMB. Therefore, the objective of this study was to develop and evaluate a novel therapeutic strategy to sensitize LMB-resistant lung cancer cells by combining LMB and doxorubicin (DOX). Among the different treatment regimens, pretreatment with DOX (pre-DOX) and subsequent treatment with LMB to A549 cells significantly decreased the 50% inhibitory concentration (IC50) as compared to that of LMB alone (4.4 nM *vs.* 10.6 nM, *P*<0.05). Analysis of cell cycle and apoptosis by flow cytometry further confirmed the cytotoxic data. To investigate molecular mechanisms for this drug combination effects, p53 pathways were analyzed by Western blot, and nuclear proteome was evaluated by two dimensional-difference gel electrophoresis (2D-DIGE) and mass spectrometry. In comparison with control groups, the levels of p53, phospho-p53 (ser15), and p21 proteins were significantly increased while phospho-p53 (Thr55) and survivin were significantly decreased after treatments of pre-DOX and LMB (*P*<0.05). The 2D-DIGE/MS analysis identified that sequestosome 1 (SQSTM1/p62) had a significant increase in pre-DOX and LMB-treated cells (*P*<0.05). In conclusion, our results suggest that drug-resistant lung cancer cells with p53 wild type could be sensitized to cell death by scheduled combination treatment of DOX and LMB through activating and restoring p53 as well as potentially other signaling pathway(s) involving sequestosome 1.

## Introduction

Lung cancer continues to be the leading cause of cancer death worldwide and in the United States [1]. Non-small-cell lung cancer (NSCLC) remains the predominant form of lung cancer (about 85% of all lung cancers), among which lung adenocarcinoma (AC) is the most frequent histologic subtype for all sexes and races combined [2]. The prognosis of lung cancer is very poor, with a 5-year survival rate of less than 15% in the United States. Chemotherapy continues to be the most frequent treatment to prolong survival and improve quality of life [3], [4], [5], [6].

Cancer chemotherapy has been used successfully in a variety of circumstances involving malignancies, however, its effectiveness has often been limited by drug resistance and side effects [7]. Therapies focusing on specific molecule(s)/pathway(s) have the potential to overcome these limitations [7]. Leptomycin B (LMB) and/or its derivatives, which can efficiently inhibit nuclear export by specifically inhibiting chromosome region maintenance 1 (CRM1), has been recognized as a novel class of cancer therapeutics [8], [9], [10], [11], [12]. CRM1, the best characterized nuclear export receptor, plays an essential role in canonical nuclear export signal (NES)-dependent nuclear export, including major tumor suppressor proteins (TSPs) such as p53, FOXO, pRB, p21, p27, etc., as well as the inhibitor of NF-κB, namely I-κB [9], [13]. Recent studies have reported that CRM1 is expressed at a significantly higher level in cervical cancer as compared to normal tissue [14] and could serve as a prognostic factor for ovarian cancer [15] and osteosarcoma [16]. Our recently published *in vitro* studies using normal lung epithelial cells [17] and a bitransgenic mouse model [18] have suggested that CRM1 plays a critical role in lung cancer development. In addition, CRM1 was over-expressed in a tobacco carcinogen-induced lung AC mouse model, and human lung AC (unpublished data). These findings suggest CRM1 could serve as a molecular target for cancer treatment, including lung cancer.

LMB is a highly specific and potent inhibitor of CRM1 function by irreversibly binding with the sulfhydryl group of a Cys residue near or within the cargo binding domain of CRM1 (alkylating Cys 528) [19], [20]. Thus, LMB could prevent cytoplasmic localization and modulate cancer-specific pathways, such as the inactivation of important tumor suppressors like p53 [10]. Our recent study demonstrated that lung AC cell line A549 (p53 wild type) was more resistant to LMB than other cell lines with the p53 mutant or null [12]. It is well known that p53 plays an important role in promoting genomic stability, cell cycle arrest, apoptosis, DNA repair, and senescence. Studies have suggested that the functions of wild type p53 on cell growth arrest and DNA repair could increase resistance to radio- or chemo- therapeutic agents; it is also prone to potentiate apoptosis in response to severe DNA damage [21], [22], [23]. Therefore, to sensitize lung cancer cell to the chemotherapeutic effect of LMB, we herein propose a therapeutic strategy combining LMB with other drugs by inducing severe DNA damage and p53 activation which could eventually lead to increased function of p53 in apoptosis rather than in DNA repair. Doxorubicin (DOX) is a widely used chemotherapeutic agent that induces apoptosis in various cancer cells through activation of p53. It has been used in the treatment of a variety of solid tumors. However, drug resistance in DOX containing regimens is a major issue which prevents better response rates and cures and cardiotoxic side effects have been reported in cancer patients treated with DOX [24], [25], [26]. Individual treatments of DOX resulted in a strong resistance in many cancer cell lines including A549, due to several mechanisms including drug bioavailability [27], [28] or NF-κB activation [29]. If DOX is combined with other chemotherapeutic drugs, lower doses may be used to not only reduce side effects, but also increase efficacy [30].

In this study, we sought to revert drug resistance to DOX and/or LMB in A549 cells via different therapeutic regimens of a co-treatment of DOX and LMB, as well as evaluate their possible molecular mechanisms. We found that pretreatment of DOX with the subsequent treatment of LMB sensitized the drug-resistant A549 cells to the chemotherapeutic effect of LMB. These changes might result from the initial activation of p53 by DOX treatment and consequently CRM1 function blocking by LMB treatment to accumulate activated p53 in the nuclear compartment. Furthermore, signaling pathways involving molecules other than p53 might also play important roles in promoting therapeutic effects of the combined treatment of DOX and LMB.

## Materials and Methods

### Reagents

Doxorubicin (DOX) and dimethylsulfoxide (DMSO) were purchased from Sigma-Aldrich Co. LLC, St. Louis, MO. LMB (1 mM) was purchased from LC Labs, Woburn, MA. The stocks of DOX (10 mg/mL) and LMB were diluted to the required concentration immediately before use with growth media. 3-(4,5-Dimethylthiazol-2-yl)-2,5-diphenyltetrazolium bromide (MTT) was purchased from USB Corporation. RPMI-1640 medium, penicillin/streptomycin, and fetal bovine serum (FBS) were purchased from Thermo scientific, Logan, UT. Primary antibodies, including p53, phospho-p53 (Ser15), phospho-p53 (Thr55), p21, sequestosome 1 (SQSTM1/p62), and survivin, were purchased from Santa Cruz Biotechnology, Santa Cruz, CA. Primary rabbit polyclonal anti-α-tubulin was purchased from Abcam, Cambridge, MA. Horseradish peroxidase (HRP)-conjugated donkey anti-rabbit IgG and an enhanced chemiluminescence (ECL) kit were purchased from GE Healthcare, Piscataway, NJ. Radioimmunoprecipitation assay (RIPA) lysis buffer was purchased from Santa Cruz Biotechnology.

### Cells and Cell Culture

Human lung adenocarcinoma epithelial cell lines A549 and NCI-H358 were obtained from American Type Culture Collection (ATCC). The cells were cultured in RPMI-1640 medium supplemented with 10% fetal bovine serum (FBS), 50 U/mL penicillin, and 50 mg/mL streptomycin. The cells were incubated at 37°C in a humidified incubator with 95% air and 5% CO_2_ by volume. Cells were sub-cultured or plated for subsequent treatment until they approached approximately 80% confluence.

### Cell Viability Assay

Cell viability was evaluated using the MTT assay as previously described [17]. Briefly, cells were plated at 5×10^3^ cells per well in 96-well plates. Based on the cytotoxicity of DOX or LMB observed in this study and previous reports [9], [12], [26], [31], [32], [33], 0.5 nM LMB or 0.5 µM DOX was selected for co-treatment or pretreatment. The cells were treated with the following: 1) DOX alone (0–5 µM) for 24 and 48 h; 2) LMB alone (0–10 nM) for 24 and 48 h; 3) co-treatment of 0.5 nM LMB and DOX (0–5 µM) simultaneously (DOX+LMB (0.5 nM)) for 24 and 48 h; 4) co-treatment of 0.5 µM DOX and LMB (0–10 nM) simultaneously (LMB+DOX (0.5 µM)) for 24 and 48 h; 5) pretreatment of 0.5 nM LMB for 24 h (pre-LMB) and subsequent DOX (0–5 µM) for 48 h (pre-LMB+DOX); and 6) pretreatment of 0.5 µM DOX for 24 h (pre-DOX) and subsequent LMB (0–5 nM) for 48 h (pre-DOX+LMB). Ethanol (EtOH, 0.1%) was used as the vehicle control for LMB. Three hours before the end of each time point, 15 µL of MTT (10 mg/mL) was added to each well and incubated at 37°C. At each time point, when purple precipitate was clearly visible under the microscope, 100 µL of 100% DMSO was added to all wells and cell viability was determined by measuring absorbance at 570 nm (reference wavelength = 630 nm) using a SpectraMax Plus Spectro-photometer (Molecular Devices, Sunnyvale, CA). Six replicates at each concentration and time point were analyzed. Experiments were performed independently in triplicate. Vehicle-treated controls and blanks were incubated in the same plate under the same conditions. Fractional absorbance was calculated by using the following formula: % cell viability = mean absorbance in test wells/mean absorbance in control wells ×100.

### Analysis of Cell Cycle and Apoptosis by Flow Cytometry

Cells were first given pretreatment of 0.5 µM DOX for 24 h and then were treated with LMB for additional 48 h. Therefore, the cells were harvested after a total of 72 h of treatment. Based on the cell viability assay, a total of 6 groups of A549 cells with different treatments were analyzed, including control, 0.5 µM DOX (pre-DOX), 1 nM LMB (LMB1), pre-DOX and 1 nM LMB (pre-DOX+LMB1), 5 nM LMB (LMB5), and pre-DOX and 5 nM LMB (pre-DOX+LMB5). For cell cycle analysis, a total of 2×10^5^ cells from each treatment group were collected and fixed in 70% ethanol for more than 24 h at 4°C. Cells were stained with Guava Cell Cycle Reagent (Millipore) and run on a Guava EasyCyte™ Flow Cytometer (Millipore). A total of 5×10^3^ events were counted, and the percentage of cells in the pre-G1, G0/G1, S, and G2/M phases of the cell cycle were determined using GuavaSoft software (Millipore). For apoptosis analysis, ViaCount assay was performed to determine viable and dead cells. In brief, the cell suspension (5×10^5^ cells/mL) was mixed with Guava ViaCount reagent (Millipore), and the mixture was incubated at room temperature for 5 minute to stain cells. The stained cell samples were run on a Guava EasyCyte™ Flow Cytometer (Millipore). A total of 5×10^3^ events were counted and data were acquired using Guava ViaCount software (Millipore). Each sample was run in triplicate and each experiment was repeated three times.

### Western Blot

The same 6 treatment groups of A549 cells as described in the flow cytometry were analyzed for Western blot. Cells in each group were lysed in RIPA lysis buffer on ice. The lysates were sonicated and then centrifuged at 13,000× g for 5 min at 4°C to collect the supernatant. Protein concentrations were measured using the Bio-Rad Bradford protein assay. A total of 30 µg of protein per sample was separated by 12% SDS-polyacrylamide gel electrophoresis (SDS-PAGE) and then transferred to polyvinylidene fluoride (PVDF) membranes. The immobilized proteins were then incubated overnight at 4°C in blocking buffer containing 3% nonfat dry milk in 1× phosphate buffered saline (PBS) and 0.1% Tween 20 (1× PBST). After blocking, the membranes were probed with the primary antibody for 1 h. Antibody binding was detected with donkey anti-rabbit IgG-HRP at a dilution of 1∶1,000 for 1 h at room temperature. After a brief incubation with ECL, the signals on membranes were exposed to X-ray films (Fujifilm Corporation, Tokyo). Relative densitometric digital analysis of protein bands were determined using Quantity One software (Bio-Rad) and normalized by the intensity of the housekeeping gene (α-Tubulin, 1∶10,000 dilution) for each sample.

### Effects of DOX and LMB on Nuclear Protein Profile

To evaluate the effects of DOX and LMB on proteins besides those in the p53 pathway, a gel-based proteomic approach, two dimensional-difference gel electrophoresis (2D-DIGE), was first performed to investigate nuclear protein profiles after LMB treatment. Protein spots showing major changes were identified by liquid chromatography mass spectrometry (LC/MS/MS) and confirmed by Western blot. Changes in protein(s) were further evaluated in cells with combined treatment of DOX and LMB by Western blot.

#### Nuclear Protein Extraction

For proteomic analysis, nuclear proteins were extracted following the protocols as described by Lu *et al* [34]. In brief, based on our previous study [12], A549 or NCI-H358 cells, treated with vehicle control (0.1% EtOH) or 20 nM LMB for 24 h (in duplicate), were rinsed with ice-cold PBS, harvested, and suspended in ice-cold Buffer A containing 10 mM tris-HCl (pH: 7.4, Bio-Rad), 8 M Urea (Bio-Rad), 4% (w/v) 3-[(3-cholamidopropyl) dimethylammonio]-1-propanesulfonate (CHAPS, Bio-Rad), 0.5 mM ethylenediaminetetraacetic acid (EDTA, Bio-Rad), 2.5 mM MgCl_2_ (EMD Chemicals, Gibbstown, NJ), 0.5 mM PMSF (Santa Cruz Biotechnology), 1× protease inhibitor cocktail (Roche, Basel, Switzerland), and 1% (v/v) NP-40 (USB Corporation, Cleveland, OH). The mixture was homogenized with a 21-gauge needle, followed by centrifuging the homogenate at 700× g for 10 min at 4°C to precipitate the nuclei. The cytoplasmic extracts in supernatants were collected and pellets were resuspended in 1 mL of ice-cold Buffer B (20 mM tris-HCl, pH 8.5 and 1× protease inhibitor cocktail), sonicated on ice, and mixed with 0.737 g urea, 0.267 g thiourea, and 0.07 g (w/v) CHAPS. After incubation on ice for 1 h, supernatants containing nuclear extracts were collected by centrifugation at 100,000× g for 1 h at 4°C. Protein concentrations were measured by the Bradford assay (Bio-Rad). The quality of nuclear extraction was determined, and the identified protein was confirmed by Western blots. α-tubulin (present in cytoplasm) and histone 3 (present in nucleus) were used to validate and confirm the purity of protein fractions.

#### 2D-DIGE

Nuclear protein extractions for 2D-DIGE were run as previously described [35]. In brief, nuclear protein extractions from A549 or NCI-H358 cells with or without LMB treatment (in duplicate) were reversely labeled with Cy3 and Cy5, respectively (GE Healthcare). Tubes containing 50 µg of each sample were combined with 1 µL of diluted Cy3 or Cy5 (400 pmol/µL in N,N-dimethylformamide, Sigma). After centrifugation, the mixture was left on ice for 30 min without light exposure. Thereafter, the reaction was stopped by the addition of 1 µL of 10 mM lysine (Sigma) and placement of samples on ice for 10 min in the dark. Samples (containing 100 µg proteins) labeled with Cy3 and Cy5, were diluted to 300 µL by adding 2D rehydration buffer (BioRad) consisting of 8 M urea, 0.5% CHAPS, 10 mM dithiothreitol (DTT, Bio-Rad), 0.2% biolytes ampholyte, and trace bromophenol blue. Samples were then applied to 17-cm immobilized linear pH 3–10 gradient (IPG) strips (BioRad) for overnight rehydration. Isoelectric focusing was conducted at 250 V for 20 min, gradually increased to 10,000 within 2.5 h, and held at 10,000 V for a total of 50,000 Voltage hours (Vh). IPG strips were subsequently equilibrated with buffer I (6 M urea, 2% SDS, 375 mM tris-HCl (pH 8.8), 20% glycerol, 130 mM DTT, and trace bromophenol blue) and buffer II (6 M urea, 2% SDS, 375 mM Tris-HCl, 20% glycerol, 135 mM iodoacetamide, and trace bromophenol blue). Proteins were then separated with 12% SDS-PAGE gels and visualized using a Typhoon Trio Imager (GE Healthcare) at excitation wavelengths of 532 and 633 nm for Cy3 and Cy5, respectively. Images were manipulated and analyzed by DeCyder and ImageQuant software (GE Healthcare); protein intensity differences were calculated for each spot on every gel.

#### In-gel Digestion

The 2D gels were stained with SYPRO-RUBY (Bio-Rad). Spots of interest were isolated using a spot picker, and placed into a 0.5 mL Eppendorf tube for trypsin digestion on a ProGest (Genomic solutions) workstation [35]. In brief, gel plugs were washed with diH_2_O, and treated with acetonitrile (ACN) for 15 min. The gel pieces were rehydrated with 10 mM DTT and 0.1 M ammonium bicarbonate (NH_4_HCO_3_) for 30 min at 60°C in water bath. Following shrinkage again with ACN, a solution containing 55 mM iodoacetamide (Bio-Rad) and 0.1 M NH_4_HCO_3_ was added for 20 min without light exposure, then replaced by 0.1 M NH_4_HCO_3_ for 15 min. The gel plugs were subsequently washed in 0.1 M NH_4_HCO_3_ for 5 min, while adding an equal volume of ACN for 5 min. After repeating the wash step twice, the gel pieces were dehydrated by ACN, and then dried for 30 min. Individual gel pieces were rehydrated in digestion buffer containing 12.5 ng/µL trypsin (Promega), 40 mM NH_4_HCO_3_, and 10% ACN at 37°C for 4 h. Formic acid was added to stop the reaction and the supernatant was analyzed directly.

#### LC/MS/MS Identification

Trypsinized peptides were analyzed by nano LC/MS/MS on a ThermoFisher LTQ Orbitrap XL. In brief, 30 µL of hydrolysate was loaded onto a 5 mm×75 µm ID C12 (Jupiter Proteo, Phenomenex) vented column at a flow-rate of 10 µL/min. Gradient elution was conducted on a 15 cm by 75 µm ID C12 column at 300 nL/min. A 30 min gradient was employed. The mass spectrometer was operated in a data-dependent mode, and the six most abundant ions were selected for MS/MS. Mass spectrometry results were searched using Mascot (www.matrixscience.com). Samples were processed in the Scaffold algorithm using DAT files generated by Mascot. Parameters for LTQ Orbitrap XL data require a minimum of 2 peptide matches per protein with minimum probabilities of 90% at the protein level.

### Statistical Analyses

Factorial ANOVA was performed to test the effects of DOX and/or LMB concentrations and incubation times on cell viability. Probit analysis was used to calculate the 50% inhibitory concentrations (IC50s). For the data obtained from flow cytometry, the average cell percentages were calculated and statistical significance was determined via one-way ANOVA and post hoc tests. For the protein expression levels among control, pre-DOX, LMB1, pre-DOX+LMB1, LMB5, and pre-DOX+LMB5, one-way ANOVA and Tukey's post hoc tests were used to compare densitometric intensity of individual samples between groups. For 2D-DIGE, image analysis was carried out with DeCyder software (GE Healthcare) and ImageMaster software. For DeCyder software, the Differential In-Gel Analysis (DIA) module was used to process a pair of images from a single gel, and perform spot detection and quantification. The Biological Variation Analysis (BVA) was employed to calculate ratios between samples and controls by performing a gel-to-gel matching of the pair of spot maps from each gel. The spots with more than a two-fold change in reverse-labeled duplicated experiments as compared with controls were considered as target proteins. All analyses were performed using SPSS software (SPSS, Inc., Chicago, IL, USA) and differences with *P*<0.05 were considered statistically significant.

## Results

### Cytotoxicity of DOX or LMB

**Figure 1:**
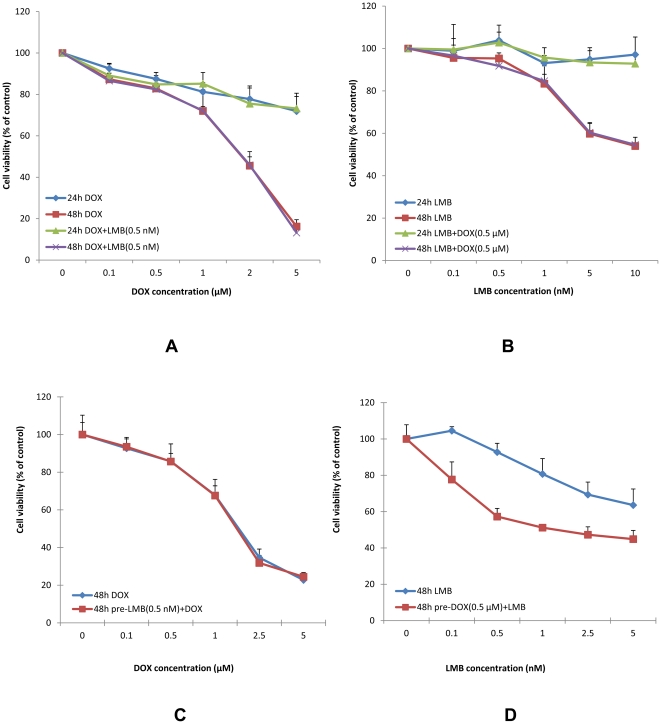
Cytotoxic effects of DOX and LMB on A549 cells. A, Cytotoxic effects of DOX alone and DOX+LMB on cell viability of A549 cells as determined by the MTT assay. Data are expressed as the percentage by comparing to vehicle control for DOX and LMB (0.5 nM) for DOX+LMB. Values are represented as means ± SD, n = 6. B, Cytotoxic effects of LMB alone and LMB+DOX on cell viability of A549 cells as determined by the MTT assay. Data are expressed as the percentage by comparing to vehicle control for LMB and DOX (0.5 µM) for LMB+DOX. Values are means ± SD, n = 6. C, Cytotoxic effects of DOX alone and pre-LMB+DOX on cell viability of A549 cells at 48 h as determined by the MTT assay. Data are expressed as the percentage by comparing to vehicle control for DOX and pre-LMB for pre-LMB+DOX. Values are means ± SD, n = 6. D, Cytotoxic effects of LMB alone and pre-DOX+LMB on cell viability of A549 cells at 48 h as determined by the MTT assay. Data are expressed as the percentage by comparing to vehicle control for LMB and pre-DOX for pre-DOX+LMB. Values are means ± SD, n = 6. Experiments performed in triplicate yielded similar results.

The MTT assay was performed to determine cell viability at each time point. As shown in Figure 1A and 1B, both DOX and LMB significantly inhibited cell proliferation of A549 in a dose- and time- dependent manner (*P*<0.001). The IC50s of DOX and LMB at 48 h were 2.2 µM and 10.6 nM, respectively (Table 1). Similarly, both DOX and LMB significantly inhibited cell proliferation of NCI-H358 in a dose- and time- dependent manner (*P*<0.001, Figure S1A and S1B).

**Table 1:** Cytotoxicity of DOX and LMB on A549 cells.

Treatment 1	Treatment 2	IC50s
DOX at different concentrations (0–5 µM)	None	2.2 µM
	0.5 nM LMB simultaneously	2.1 µM
	0.5 nM LMB 24 h earlier	2.8 µM
LMB at different concentrations (0–5 nM)	None	10.6 nM
	0.5 µM DOX simultaneously	10.4 nM
	0.5 µM DOX 24 h earlier	4.4 nM*

* *P*<0.05 in comparison to LMB alone or LMB+0.5 µM DOX simultaneously.

### Cytotoxicity of Co-treatment of DOX and LMB

Similar to DOX or LMB groups, DOX+LMB (0.5 nM) or LMB+DOX (0.5 µM) inhibited A549 proliferation in a dose- and time- dependent manner (*P*<0.001, Figure 1A and 1B). However, the simultaneous treatments of DOX+LMB (0.5 nM) or LMB+DOX (0.5 µM) did not change the cytotoxic effects on A549 cells as compared to DOX alone or LMB alone at both 24 and 48 h (*P*>0.05, Figure 1A and 1B). The IC50s of DOX+LMB (0.5 nM) and LMB+DOX (0.5 µM) at 48 h were 2.1 µM and 10.4 nM, respectively (Table 1). Similarly, the simultaneous treatments of DOX+LMB (0.5 nM) or LMB+DOX (0.5 µM) did not change the cytotoxic effects on NCI-H358 cells as compared to DOX alone or LMB alone at both 24 and 48 h (*P*>0.05, Figure S1A and S1B).

### Cytotoxicity of pre-LMB+DOX or pre-DOX+LMB

As shown in Figure 1C, pretreatment of 0.5 nM LMB did not boost the cytotoxic effects of DOX on A549 cells at 48 h as compared with DOX alone (*P*>0.05). The IC50s at 48 h of pre-LMB+DOX and DOX alone were 2.8 and 2.2 µM, respectively (Table 1). However, the pretreatment of 0.5 µM DOX significantly increased the cytotoxic effect of LMB on A549 cells at 48 h (*P*<0.05, Figure 1D). The IC50 at 48 h of pre-DOX+LMB was 4.4 nM, which was significantly lower than that of LMB alone (10.6 nM, *P* = 0.037, Table 1). Furthermore, either pre-LMB or pre-DOX did not improve the cytotoxic effects of DOX or LMB on NCI-H358 cells (*P*>0.05, Figure S1C and S1D).

### Effects of DOX and LMB on Cell Cycle and Apoptosis

**Figure 2:**
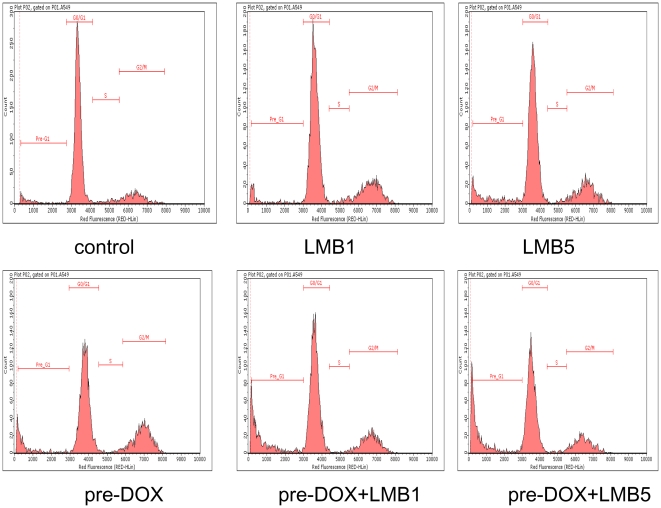
Flow cytometry analyses of cell cycle and apoptosis in A549 cells after DOX and LMB treatment. Representative histograms of cell cycle analyses in DOX and LMB-treated A549 cells. Control, pre-DOX, LMB1, pre-DOX+LMB1, LMB5, and pre-DOX+LMB5 were harvested and labeled with Guava Cell Cycle Reagent (Millipore) and analyzed by flow cytometry (pre-G1, G0/G1, S, and G2/M). The y-axis shows the number of cells counted and the x-axis shows an increasing amount of Guava Cell Cycle Reagent incorporation/cell (left to right). Experiments performed in triplicate yielded similar results. LMB1: 1 nM LMB, LMB5: 5 nM LMB.

Cell proliferation inhibition could be the result of either cell cycle arrest or apoptosis, thus these two aspects were further examined by flow cytometry analysis of A549 cells after LMB and DOX treatment. The cell cycle analysis revealed that the percentage of cells in G2/M were 15.1±0.4, 26.9±2.8, 22.7±1.0, 22.9±4.2, 22.5±2.8, and 18.6±1.3 in the control, pre-DOX, LMB1, pre-DOX+LMB1, LMB5, and pre-DOX+LMB5, respectively (Table 2). Pre-DOX, LMB1, pre-DOX+LMB1, LMB5, and pre-DOX+LMB5 all resulted in an accumulation in the G2/M phase versus control (*P*<0.05, Figure 2 and Table 2). In addition, the cell cycle analysis revealed that the percentage of cells in pre-G1 were 5.4±2.2, 9.0±2.1, 8.2±2.0, 18.6±7.1, 10.2±4.7, and 27.5±2.8 in the control, pre-DOX, LMB1, pre-DOX+LMB1, LMB5, and pre-DOX+LMB5, respectively (Table 2). Pre-DOX+LMB1 and pre-DOX+LMB5 resulted in a definitive accumulation in the pre-G1 phase versus not only control but also LMB alone (*P*<0.01, Figure 2 and Table 2). Analysis of apoptosis revealed that LMB treatment significantly induced cell apoptosis (*P*<0.01, Table 2). Apoptosis was further increased after cells were co-treated with pre-DOX and LMB compared with LMB alone (*P*<0.01, Table 2). The percentage of apoptotic cells were 13.2±1.6, 15.8±2.6, 19.2±2.4, 27.1±0.6, 22.4±4.0, and 29.6±2.1 in the control, pre-DOX, LMB1, pre-DOX+LMB1, LMB5, and pre-DOX+LMB5, respectively (Table 2).

**Table 2:** Effects of DOX and LMB on cell cycle and apoptosis of A549 cells.

	Cell Cycle (%)				Apoptosis (%)
	Pre-G1	G0/G1	S	G2/M	
Control	5.4±2.2	74.5±2.9	5.0±0.9	15.1±0.4	13.2±1.6
Pre-DOX	9.0±2.1	61.6±1.7**	2.5±0.7**	26.9±2.8**	15.8±2.6
LMB1	8.2±2.0	66.6±1.5**	2.5±0.1**	22.7±1.0**	19.2±2.4**
Pre-DOX+LMB1	18.6±7.1** ##	57.2±2.8** ##	1.2±0.1** #	22.9±4.2**	27.1±0.6** ##
LMB5	10.2±4.7	65.0±1.7**	2.3±0.3**	22.5±2.8**	22.4±4.0**
Pre-DOX+LMB5	27.5±2.8** ##	52.5±1.9** ##	1.5±0.2** #	18.6±1.3* #	29.6±2.1** ##

* *P*<0.05 in comparison to control;

** *P*<0.01 in comparison to control.

# *P*<0.05 in comparison to LMB alone;

## *P*<0.01 in comparison to LMB alone.

### Western Blot Analyses of p53, Phospho-p53 (Ser15), Phospho-p53 (Thr55), p21, and Survivin Protein Expression after DOX and LMB Treatment

**Figure 3:**
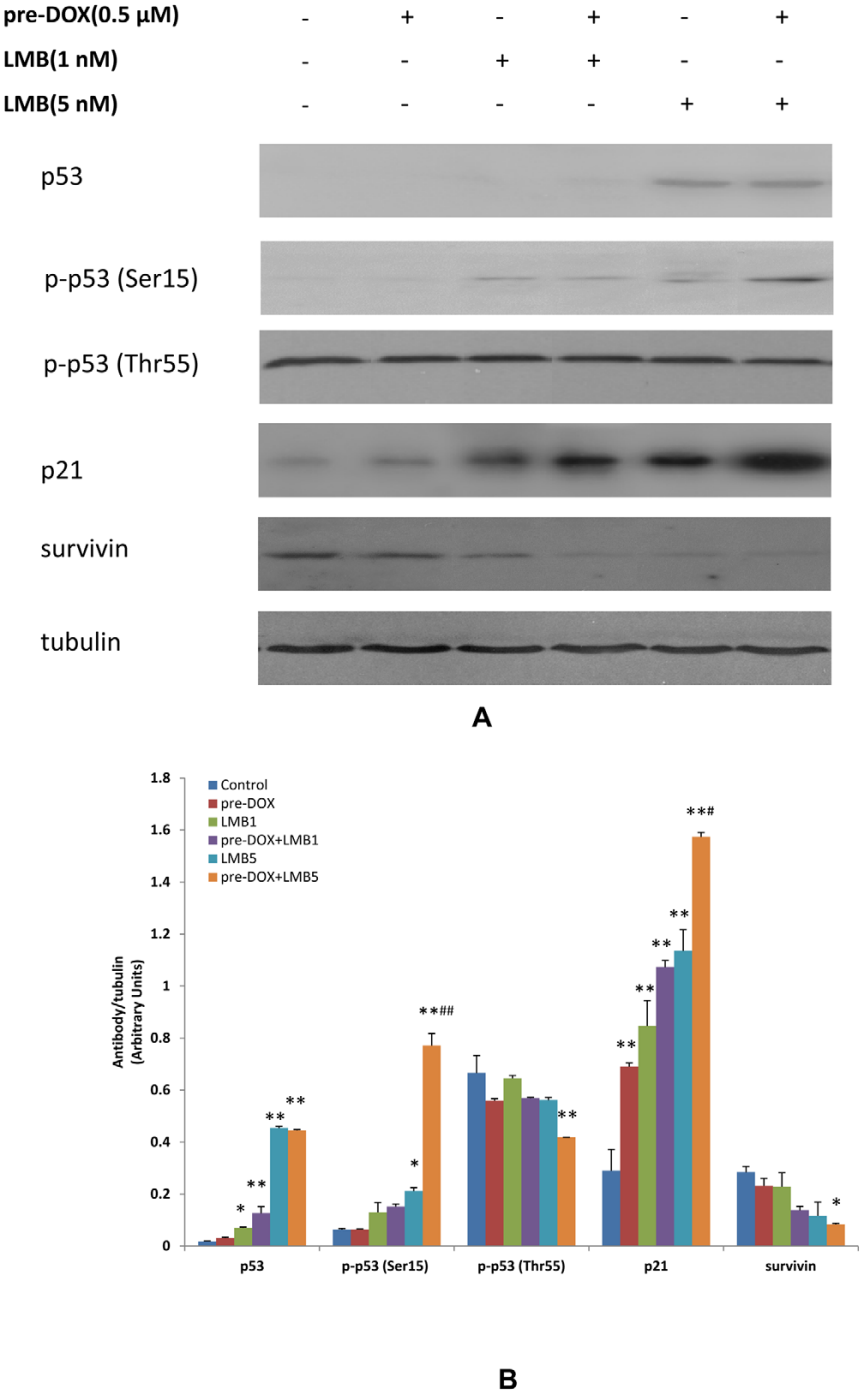
Western blot analyses of protein expression in A549 cells after DOX and LMB treatment. A, Effects of pre-DOX+LMB treatment on the protein expression of p53, phospho-p53 (Ser15), phospho-p53 (Thr55), p21, and survivin in A549. Cells were treated with 0.5 µM DOX 24 h before treatment with LMB (1 nM or 5 nM). After 48 h LMB treatment, cells were harvested for Western blot analysis to determine protein levels. Blots were also probed for α-tubulin to confirm equal protein loading. B, The relative protein intensities of p53, phospho-p53 (Ser15), phospho-p53 (Thr55), p21, and survivin as compared with the intensity of α-tubulin. The intensity of each band was quantified using Quantity One software. Data are means ± SD, n = 3. The experiments were conducted in triplicate. LMB1: 1 nM LMB; LMB5: 5 nM LMB; *, *P*<0.05 compared to control; **, *P*<0.01 compared to control; #, *P*<0.05, compared to LMB5; ##, *P*<0.01, compared to LMB5.

Expression levels of p53, phospho-p53 (Ser15), and p21 (a downstream target of p53) were significantly increased in cells treated with pre-DOX+LMB than those of controls and showed a significant dose-response effect (Figure 3A). The relative protein expression levels of p53 (arbitrary units) were 0.02±0.00, 0.03±0.00, 0.07±0.00, 0.13±0.03, 0.45±0.01, and 0.44±0.00 in the control, pre-DOX, LMB1, pre-DOX+LMB1, LMB5, and pre-DOX+LMB5, respectively (Figure 3B, *P*<0.05, LMB1 *vs.* control; *P*<0.01, pre-DOX+LMB1, LMB5, or pre-DOX+LMB5 *vs.* control). The relative protein expression levels of phospho-p53 (Ser15) (arbitrary units) were 0.06±0.00, 0.06±0.00, 0.13±0.03, 0.15±0.01, 0.21±0.01, and 0.77±0.04 in the control, pre-DOX, LMB1, pre-DOX+LMB1, LMB5, and pre-DOX+LMB5, respectively (Figure 3B, *P*<0.05, LMB5 *vs.* control; *P*<0.01, pre-DOX+LMB5 *vs.* control). Furthermore, the up-regulation of phospho-p53 (Ser15) in pre-DOX+LMB5 was significant compared with the LMB5 group (*P*<0.01). Relative protein expression levels of p21 (arbitrary units) were 0.29±0.08, 0.69±0.01, 0.85±0.09, 1.07±0.03, 1.14±0.08, and 1.57±0.02 in the control, pre-DOX, LMB1, pre-DOX+LMB1, LMB5 and pre-DOX+LMB5, respectively (Figure 3B, *P*<0.01, pre-DOX, LMB1, pre-DOX+LMB1, LMB5, and pre-DOX+LMB5 *vs.* control). Furthermore, the up-regulation of p21 in pre-DOX+LMB5 was significant (*P*<0.05) compared with the LMB5 group.

Contrary to p53, phospho-p53 (Ser15), and p21 expression levels, phospho-p53 (Thr55) and survivin (another downstream target of p53) expression levels were significantly and dose dependently decreased in cells treated with pre-DOX+LMB compared to those of controls (Figure 3A). The relative protein expression levels of phospho-p53 (Thr55) (arbitrary units) were 0.67±0.06, 0.56±0.01, 0.65±0.01, 0.57±0.00, 0.56±0.01, and 0.42±0.00 in the control, pre-DOX, LMB1, pre-DOX+LMB1, LMB5, and pre-DOX+LMB5, respectively (Figure 3B, *P*<0.01, pre-DOX+LMB5 *vs.* control). The relative protein expression levels of survivin (arbitrary units) were 0.28±0.02, 0.23±0.03, 0.23±0.05, 0.14±0.01, 0.12±0.05, and 0.08±0.00 in the control, pre-DOX, LMB1, pre-DOX+LMB1, LMB5, and pre-DOX+LMB5 treated groups, respectively (Figure 3B, *P*<0.05, pre-DOX+LMB5 *vs.* control).

### Effects of DOX and LMB on Nuclear Protein Profile

#### Nuclear Protein Extraction

**Figure 4:**
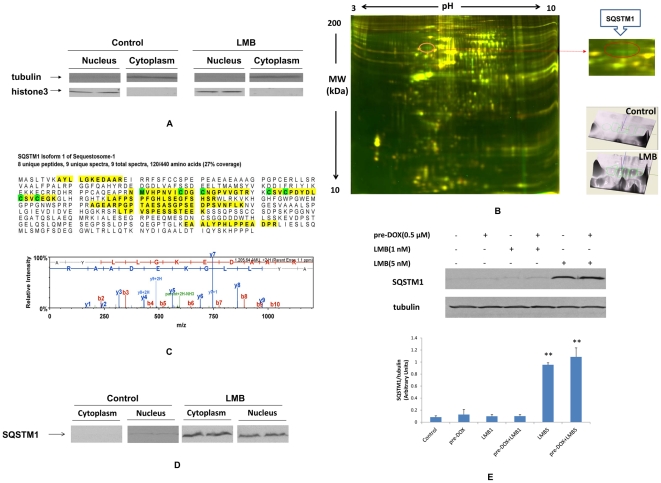
Nuclear proteome profiling in A549 cells after DOX and/or LMB treatment. A, Western blot of nuclear and cytoplasmic protein extractions from A549; α-tubulin served as an internal control for cytoplasmic proteins, and histone 3 served as a control for nuclear proteins. B, 2D-DIGE analyses of nuclear proteins in A549 cells and 3D views of SQSTM1 in A549 cell with vehicle control or LMB treatment. Nuclear proteins treated with LMB or vehicle control were labeled with Cy3 (green channel) and Cy5 (red channel), respectively. Nuclear proteins were separated based on isoelectric point (PI, horizontal axis) and molecular weight (MW, vertical axis). Approximately 1,000 protein spots were detected in nuclear extractions of A549 cells. Spots labeled with red color indicate decreased expression after LMB treatment, while spots labeled with green color indicate increased expression after LMB treatment (left panel). Magnification of 5 protein spots (right upper panel) and 3D view of vehicle control and LMB treated (right bottom panel) (identified by LC/MS/MS as sequestosome 1 (SQSTM1/p62)). C, Protein sequence and tandem mass spectrometry identification of SQSTM1. The MS/MS fragmentation spectrum (obtained after trypsin digestion) of AYLLGKEDAAR for SQSTM1 is shown. The resultant MS/MS data were processed using Mascot. D, Western blot analysis of SQSTM1 in cytoplasm and nucleus of A549 cells after LMB treatment. E, Effects of LMB alone or pre-DOX+LMB treatment on protein expression of SQSTM1 in A549 cells. The relative protein intensity of SQSTM1 was compared with the intensity of corresponding α-tubulin. The intensity of each band was quantified using Quantity One software. Data are means ± SD. Experiments were conducted in triplicate. LMB1: 1 nM LMB; LMB5: 5 nM LMB; **, *P*<0.001 compared to control.

Purity of the nuclear and cytoplasmic proteins was tested using Western blot analysis with anti-histone 3 and anti-α-tubulin. Majority of α-tubulin was found only in the cytoplasmic fraction from A549 and NCI-H358 cells; histone 3 was found only in the nuclear fraction from A549 and NCI-H358 cells, suggesting that the preparation was enriched for nuclear proteins (Figures 4A and S2A).

#### 2D-DIGE

Approximately 1,000 protein spots were detected in nuclear extractions of A549 cells. Duplicate DIGE gels with reverse label were run and showed more than 99% between-gel reproducibility. From these detected spots, 13 spots showed more than or equal to two-fold increase in LMB-treated A549 cells, out of that 5 spots with the highest changes had almost the same molecular weight (MW, approximate 60 kDa) but different isoelectric point (PI), suggesting the possibility of post-translational modifications (PTMs) of the same protein (Figure 4B). On the other hand, among the approximate 1,000 protein spots detected in nuclear extraction of NCI-H358 cells, no proteins spots showed significant changes (Figure S2B).

#### Protein Identification by LC/MS/MS and Western Blot Analysis of SQSTM1

Among the total 13 spots of interest, only 3 spots were visible by SYPRO-RUBY staining. The visible spots were among the earlier 5 spots with the same MW but different PI and had the highest changes. They were identified as SQSTM1 by LC/MS/MS. For instance, there were 9 matched peptides (27% sequence coverage) from the LC/MS/MS for the spot with the most hits (Figure 4C). To confirm and validate the results of 2D-DIGE/MS, Western blot analysis was performed. The expression of SQSTM1 in LMB-treated A549 cells was significantly increased in comparison with control cells in both nucleus and cytoplasm of cells (Figure 4D). However, SQSTM1 in LMB-treated NCI-H358 cells was not changed in both nucleus and cytoplasm of cells (Figure S2C).

#### Effects of DOX and LMB on SQSTM1 Protein Expression

Western blot analysis was performed to analyze SQSTM1 protein expression level (Figure 4E). The relative protein expression levels of SQSTM1 (arbitrary units) were 0.08±0.01, 0.13±0.06, 0.10±0.02, 0.10±0.02, 0.95±0.02 and 1.09±0.10 in the control, pre-DOX, LMB1, pre-DOX+LMB1, LMB5 and pre-DOX+LMB5, respectively (Figure 4E). The up-regulations of SQSTM1 in LMB5 and pre-DOX+LMB5 treated cells were significant compared with control cells (*P*<0.001). In addition, SQSTM1 was slightly but not significantly higher in pre-DOX+LMB5 than that of LMB5 alone.

## Discussion

LMB and/or its derivatives have been recognized as a novel class of cancer therapeutics through highly specific and potent inhibition of CRM1, a NES-dependent nuclear exporter [8], [9], [10], [11], [12], [19], [20]. Our previous study found that LMB could significantly inhibit cell proliferation of various lung AC cell lines compared to normal lung epithelial cells [12]. However, A549 cells (with p53 wild type) were more resistant to doses that were effective in other lung AC cell lines and the IC50 of LMB on A549 was close to that of normal lung epithelial cells. Combination chemotherapy could increase the therapeutic efficacy, decrease toxicity to normal cells with lower dosage, and minimize or delay the development of drug resistance. DOX is another cancer therapeutic drug, however, lung AC cells with p53 wild type, such as A549 cells, are resistant to this drug [28]. Due to DOX's specific molecular activity including p53 up-regulation and/or activation mediated apoptosis [24], [26], [31], it was used in this study to test its efficiency in combination with LMB treatment to sensitize the drug resistance of A549 to the chemotherapeutic effect of LMB. Our results for the first time report increased drug efficacy from the combined therapy of an initial DOX treatment and subsequent LMB treatment in A549 cells, but not in p53 null NCI-H358 lung AC cells. The findings of this study also revealed that simultaneous treatment of LMB and DOX, or pretreatment of LMB with subsequent treatment of DOX was not effective in A549 cells (similar in p53 null NCI-H358 lung AC cells). These results indicate that pretreatment with DOX is required for chemotherapeutic inhibition of lung AC cells and the interaction between DOX and LMB is highly schedule dependent. Furthermore, our results reveal that the molecular mechanisms involving p53 activation and other signaling protein(s)/pathway(s) involving sequestosome 1 could be the pre-requisite trigger to the observed effectiveness of combination therapy.

As expected, we found that both DOX and LMB have significant inhibitory effects on A549 cells in a dose- and time- dependent manner. The IC50 value of DOX observed in this study was comparable to previous reports that showed IC50s of 1–5 µM in A549 cells [32], [36]. Among the four regimens tested, only pre-DOX treatment could boost the cytotoxic effect of LMB, in which the IC50 decreased more than 2-fold. The total duration of sequential treatment of pre-DOX+LMB or pre-LMB+DOX was 72 h that includes 24 h of pretreatment and subsequent 48 h of co-exposure. The doses chosen for pre-LMB or pre-DOX did not show significant cytotoxic effects to A549 cells at 24–72 h. Nevertheless, the sequential treatment data were analyzed by using pre-LMB or pre-DOX as controls with their respective durations of exposure to exclude the potential cytotoxic effects from pre-LMB or pre-DOX. In addition, co-treatment of LMB and DOX for 72 h did not boost the cytotoxic effects of either LMB or DOX for both A549 and H358 cells (data not shown). Results from flow cytometry analysis further validated the cytotoxicity data. Both DOX and LMB treatment decreased the fraction of cells in G0/G1 and S phases while they increased the fraction of cells in G2/M phases, suggesting G2/M arrest of DOX/LMB-treated A549 cells. These observations are consistent with previous findings of DOX induced predominant G2 arrest [37] and LMB producing reversible G1 and G2 arrest [38]. Interestingly, although the number of cells in G2/M phase was not changed in cells treated with pre-DOX and LMB compared to LMB alone, the number of cells at pre-G1 phase was significantly increased in A549 cells treated with pre-DOX and LMB compared to LMB alone. Furthermore, the number of apoptotic cells was also significantly increased in cells treated with pre-DOX and LMB compared to LMB alone. Together, these data suggest pre-DOX treatment enhanced or facilitated the effect of LMB on mitotic arrest. Individual treatments by DOX itself caused predominantly G2/M arrest while pre-DOX and LMB induced predominantly apoptosis. In addition, apoptosis increased with increasing concentrations of LMB.

DOX is generally classified as a topoisomerase II inhibitor that induces DNA double-strand breaks. DOX, although not frequently used in recent lung cancer protocols but commonly used to treat other cancers such as leukemias, lymphomas, as well as other solid tumors [29], [33], [39]. The cellular response to DNA damage, which includes nuclear accumulation of p53, has been studied extensively using DOX [33]. Thus, the better understanding of the combination effects of DOX with other potential targeted chemotherapies, such as LMB, will lead a significant clinical milestone which can eventually overcome drug resistance. Our previous study has also suggested that p53 signaling pathway was activated after LMB treatment in A549 cells [12]. p53 can be activated through post-translational modifications such as phosphorylation, as well as subcellular localization. The phosphorylation sites have been identified at multiple locations on both N-terminus and C-terminus of p53 [40]. It is recognized that an early critical event in the stabilization and activation of p53 in response to genotoxicity is the phosphorylation of Ser15 through activation of ATM in response to DNA damage [41]. In the present study, Western blot data demonstrated that LMB was very effective in induction of both total p53 and p53 phosphorylation at Ser15 compared to the control. In addition, phospho-p53 at Ser15 was increased in cells treated with pre-DOX and LMB (5 nM) compared to LMB (5 nM) alone. Nuclear export of p53 is mediated by CRM1; this can be abrogated by LMB [9], [11], [12]. Thr55 is another important phosphorylation site for p53 function because of its location at the amino acid 43–63 residues of p53 that contain an apoptotic and growth suppression domain [42], [43]. In other studies, inhibition of Thr55 phosphorylation of p53 restored its nuclear localization and sensitized cancer cells to DNA damage [44]; the phosphorylation of Thr55 led to p53 degradation and a decrease in G1 arrest of the cell cycle [45]. The decreased phospho-p53 (Thr55) after pre-DOX and LMB treatment observed in this study was in agreement with these previous findings and was further confirmed by data on cell viability and cell cycle/apoptosis. Data from ours and other published studies have shown that LMB could increase the activation/stabilization/nuclear accumulation of p53 by blocking its nuclear export through CRM1 [9], [12], [46], [47], [48], [49], [50]. This could further lead to the increased expression of p53 downstream target genes, such as p21 [12], [49], [50]. It has also been reported that phosphorylated Ser15 of p53 localized in the nucleus [51]. Moreover, consistent with these previous findings, we also observed the p53 and phospho-p53 (Ser15) accumulated in the nuclear compartment after LMB treatment as determined by western blot analysis using nuclear/cytoplasmic protein fractions from A549 cells (data not shown). Besides LMB, DOX treatment could also induce nuclear accumulation of p53 [31], [52], [53]. Taken together, these evidences suggested that the superior cytotoxic effect of pre-DOX+LMB could be attributed to nuclear accumulation of p53. In addition, our results of p53 expression also suggest that phosphorylation on Ser15 and Thr55 sites of p53 may cooperatively regulate the stability of p53 and thereby more effectively activate p53 in response to DOX and LMB treatment. The treatment regimen of pretreatment of DOX and LMB, but not DOX and LMB simultaneously or pretreatment of LMB and subsequent DOX, could induce and activate p53 in the function of apoptosis rather than DNA repair that led to the drug sensitization of A549 cells.

The regulation of protein expression of p53 target genes involved in cell growth suppression and apoptosis was also observed after DOX and LMB treatment. For example, p21, a downstream target of p53, was elevated at the protein level after DOX and LMB treatment, especially in cells treated with pre-DOX and LMB. This elevated level of p21 could result in hypophosphorylation of the Rb protein, which in turn binds with E2F transcription factor and subsequently blocks the cell cycle [54], [55]. Besides p53, survivin expression was significantly repressed after LMB treatment, especially when pretreatment of DOX was applied before LMB. Survivin, a member of the inhibitor of apoptosis family of proteins, is negatively regulated by wild type p53 and plays an important role in regulation of both apoptosis and cell division [56]. Survivin repression caused by DNA damage may decide whether the damaged cells would die before DNA repair is accomplished by activating the p53-dependent G2/M checkpoint [57]. Moreover, nuclear export of p21 and survivin is CRM1-mediated [58], [59]. Thus, LMB may directly or indirectly modulate the expression of p21 and survivin. Collectively, the elevated level of p21 and repression of survivin were consistent with the cytotoxicity, cell cycle/apoptosis, and p53 activation after DOX and LMB treatment. The combined therapy of an initial DOX treatment (for activation of p53) and subsequent LMB treatment (for blocking CRM1 function to increase and accumulate activated steady-state level of p53 in the cellular nucleus) might be one reason for the increased effectiveness.

SQSTM1 (p62) had been identified by a proteomic approach using 2D-DIGE and MS as a possible new protein(s)/pathway(s) that could be targeted by LMB treatment in p53 wild type A549 cells but not p53 null NCI-H358 cells. SQSTM1 was first described in 1995 as a phosphotyrosin-independent ligand of the src homology 2 (SH2) domain of the lymphoid-specific src family tyrosine kinase p56Ick [60]. SQSTM1 was recently shown to be continuously shuttled between the cytoplasm and nucleus at a high rate [61]. This process is regulated by several mechanisms, such as self-interaction, polymerization, phosphorylation, aggregation, and binding to ubiquitinated targets [61]. In fact, nuclear accumulation of SQSTM1 was observed in Hela cells treated with LMB [62]. Alternatively, SQSTM1 was shown to be a negative regulator of the ras signaling pathway [60]. Since A549 contains K-ras mutation, the increase and nuclear accumulation of SQSTM1 in A549 cells after LMB treatment might further inactivate functional K-ras that resulted in cell growth inhibition. Until recently, the function of SQSTM1 in the nucleus has been rarely addressed. It has been suggested that nuclear SQSTM1 could be directly associated with chromatin [62], or play a role in regulating gene transcription [63]. SQSTM1 has been reported to interact with p53; the accumulation of SQSTM1 could slow the clearance of short lived ubiquitin-proteasome system specific substrates, such as p53 [64]. Nuclear accumulation of proteins as observed in LMB-treated A549 cells, especially when pre-DOX was added, suggests that DOX and LMB may lead to nuclear sequestration of CRM1 cargo proteins, such as SQSTM1, in regulating cell growth/proliferation/apoptosis.

In summary, the present study found that combination therapy of pretreatment with DOX followed by LMB treatment significantly increased the efficacy of LMB through p53 and potentially other molecular pathways involving sequestosome 1. Future studies of other molecular mechanisms as well as CRM1 mutations/instability/integrity are necessary to further elucidate the usefulness of LMB and/or its derivatives for clinical application. Nevertheless, our data have essential predictive and therapeutic implications that could provide a promising basis for preclinical and/or clinical trials on lung cancer treatment.

## Supporting Information

Figure S1 **Cytotoxic effects of DOX and LMB on NCI-H358 cells.** A, Cytotoxic effects of DOX alone and DOX+LMB on cell viability of NCI-H358 cells as determined by the MTT assay. Data are expressed as the percentage by comparing to vehicle control for DOX and LMB (0.5 nM) for DOX+LMB. Values are represented as means ± SD, n = 6. B, Cytotoxic effects of LMB alone and LMB+DOX on cell viability of NCI-H358 cells as determined by the MTT assay. Data are expressed as the percentage by comparing to vehicle control for LMB and DOX (0.5 µM) for LMB+DOX. Values are means ± SD, n = 6. C, Cytotoxic effects of DOX alone and pre-LMB+DOX on cell viability of NCI-H358 cells at 48 h as determined by the MTT assay. Data are expressed as the percentage by comparing to vehicle control for DOX and pre-LMB for pre-LMB+DOX. Values are means ± SD, n = 6. D, Cytotoxic effects of LMB alone and pre-DOX+LMB on cell viability of NCI-H358 cells at 48 h as determined by the MTT assay. Data are expressed as the percentage by comparing to vehicle control for LMB and pre-DOX for pre-DOX+LMB. Values are means ± SD, n = 6. Experiments performed in triplicate yielded similar results.
(TIF)

Figure S2 **Nuclear proteome profiling in NCI-H358 cells after DOX and/or LMB treatment.** A, Western blot of nuclear and cytoplasmic protein extractions from NCI-H358; α-tubulin served as an internal control for cytoplasmic proteins, and histone 3 served as a control for nuclear proteins. B, 2D-DIGE analyses of nuclear proteins in NCI-H358 cells with vehicle control or LMB treatment. Nuclear proteins treated with LMB or vehicle control were labeled with Cy3 (green channel) and Cy5 (red channel), respectively. Nuclear proteins were separated based on isoelectric point (PI, horizontal axis) and molecular weight (MW, vertical axis). Approximately 1,000 protein spots were detected in nuclear extractions of NCI-H358 cells. Spots labeled with red color indicate decreased expression after LMB treatment, while spots labeled with green color indicate increased expression after LMB treatment. C, Western blot analysis of SQSTM1 in cytoplasm and nucleus of NCI-H358 cells after LMB treatment.
(TIF)
