# Supplementary figures and images for: Chemotherapeutic Sensitization of Leptomycin B Resistant Lung Cancer Cells by Pretreatment with Doxorubicin
Source: PLoS One. 2012 Mar 7;7(3):e32895. doi: 10.1371/journal.pone.0032895 (PMC3296751; doi:10.1371/journal.pone.0032895)

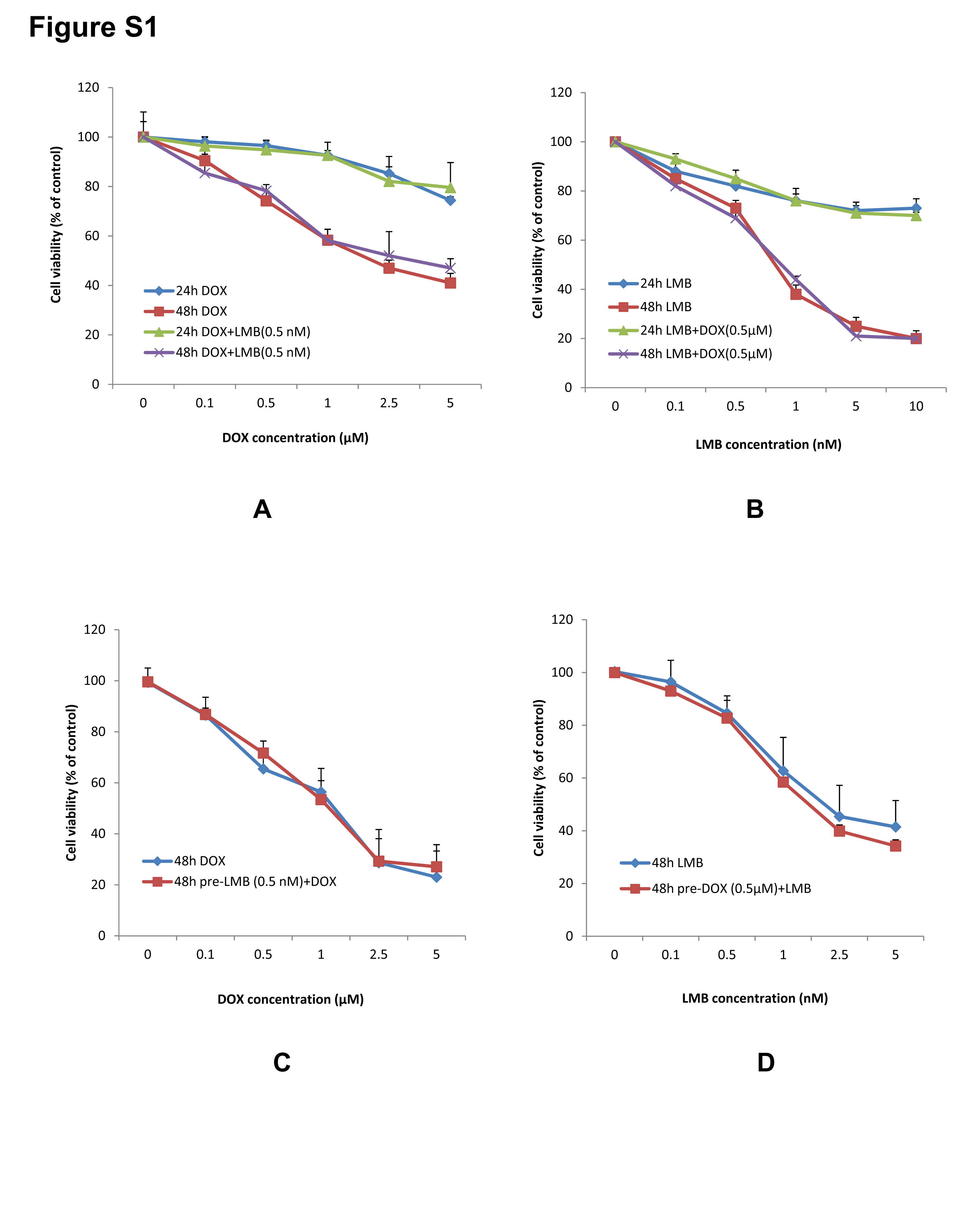

Supplement: Figure S1 — Cytotoxic effects of DOX and LMB on NCI-H358 cells. A, Cytotoxic effects of DOX alone and DOX+LMB on cell viability of NCI-H358 cells as determined by the MTT assay. Data are expressed as the percentage by comparing to vehicle control for DOX and LMB (0.5 nM) for DOX+LMB. Values are represented as means ± SD, n = 6. B, Cytotoxic effects of LMB alone and LMB+DOX on cell viability of NCI-H358 cells as determined by the MTT assay. Data are expressed as the percentage by comparing to vehicle control for LMB and DOX (0.5 µM) for LMB+DOX. Values are means ± SD, n = 6. C, Cytotoxic effects of DOX alone and pre-LMB+DOX on cell viability of NCI-H358 cells at 48 h as determined by the MTT assay. Data are expressed as the percentage by comparing to vehicle control for DOX and pre-LMB for pre-LMB+DOX. Values are means ± SD, n = 6. D, Cytotoxic effects of LMB alone and pre-DOX+LMB on cell viability of NCI-H358 cells at 48 h as determined by the MTT assay. Data are expressed as the percentage by comparing to vehicle control for LMB and pre-DOX for pre-DOX+LMB. Values are means ± SD, n = 6. Experiments performed in triplicate yielded similar results. (TIF) [file pone.0032895.s001.tif]

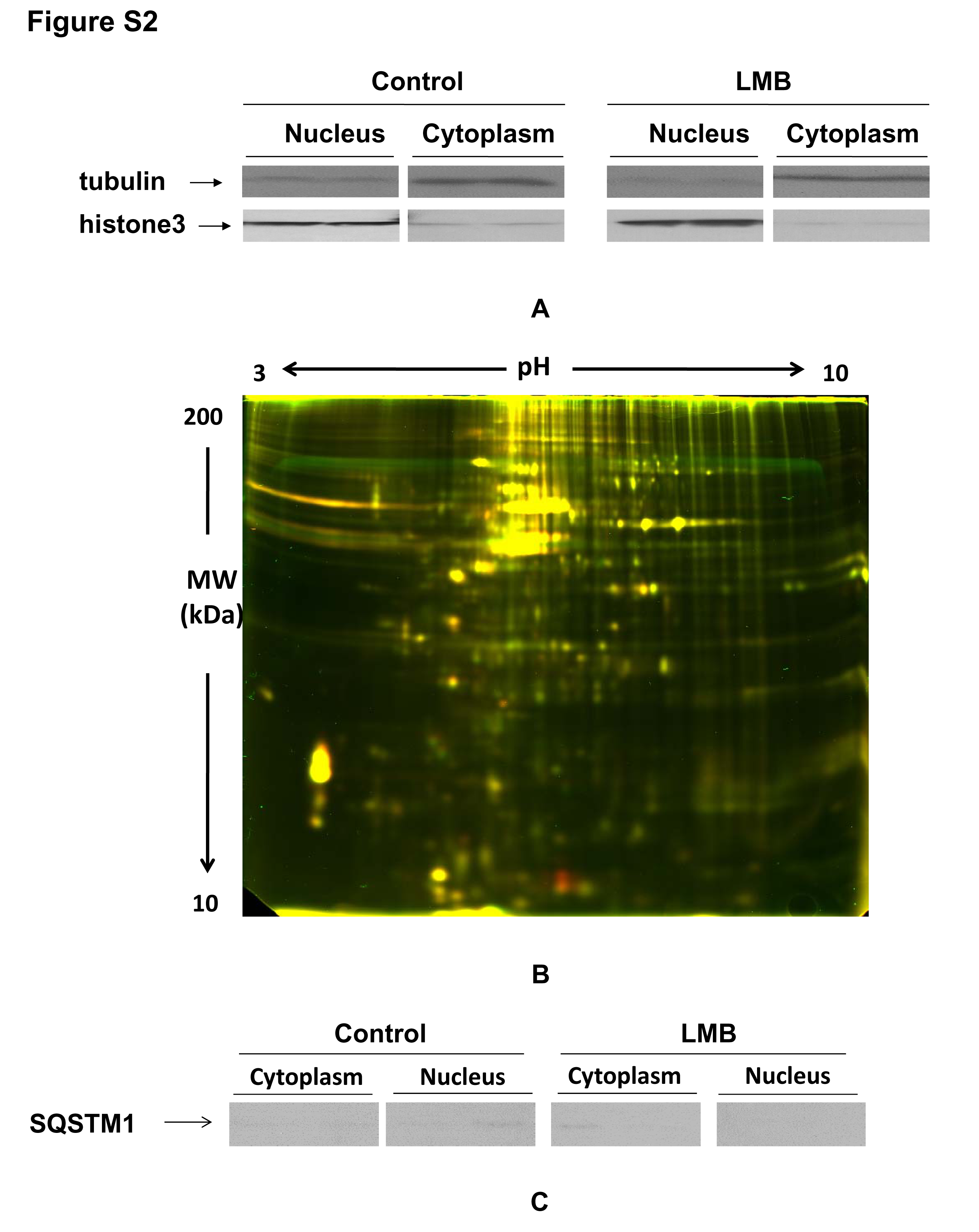

Supplement: Figure S2 — Nuclear proteome profiling in NCI-H358 cells after DOX and/or LMB treatment. A, Western blot of nuclear and cytoplasmic protein extractions from NCI-H358; α-tubulin served as an internal control for cytoplasmic proteins, and histone 3 served as a control for nuclear proteins. B, 2D-DIGE analyses of nuclear proteins in NCI-H358 cells with vehicle control or LMB treatment. Nuclear proteins treated with LMB or vehicle control were labeled with Cy3 (green channel) and Cy5 (red channel), respectively. Nuclear proteins were separated based on isoelectric point (PI, horizontal axis) and molecular weight (MW, vertical axis). Approximately 1,000 protein spots were detected in nuclear extractions of NCI-H358 cells. Spots labeled with red color indicate decreased expression after LMB treatment, while spots labeled with green color indicate increased expression after LMB treatment. C, Western blot analysis of SQSTM1 in cytoplasm and nucleus of NCI-H358 cells after LMB treatment. (TIF) [file pone.0032895.s002.tif]
